# Descriptive Evidence of COVID-19’s Impact on Black LGBTQ Adults Amidst Ongoing Health Inequities

**DOI:** 10.1093/geroni/igab046.3686

**Published:** 2021-12-17

**Authors:** Charleigh Flohr, Cassandra Burton

**Affiliations:** 1 Human Rights Campaign Foundatiom, Washington, District of Columbia, United States; 2 AARP, Atlanta, Georgia, United States

## Abstract

Black LGBTQ people significant challenges and discrimination as they face the barriers of living at the intersection of multiply marginalized identities, which have worsened during the coronavirus pandemic. At a baseline, LGBTQ people exhibit elevated risk of being negatively impacted by the pandemic across health, economic and other social outcomes (Cahill et al., 2020; Heslin & Hall, 2021; Human Rights Campaign, 2020). Black LGBTQ people have also suffered significant economic losses (Human Rights Campaign Foundation, 2020) . Methods: The Human Rights Campaign Foundation and AARP supported and partnered with Community Marketing & Insights to conduct a survey of 1,815 Black LGBTQ adults in the United States. The online survey was fielded between September 21 and October 30, 2020. Many Black LGBTQ adult participants report healthcare discrimination in the last three years, with 19% reporting racial discrimination and 11% reporting sexual orientation-based discrimination. Furthermore, 31% of Black transgender adults report healthcare discrimination in the last three years. Preventing or treating COVID-19 is an important health concern to 67% of Black LGBTQ adults aged fifty-five and older, 63% of Black LGBTQ adults aged thirty-five to fifty-four and 53% aged eighteen to thirty-four. Overall, Black LGBTQ adults report being significantly impacted by the pandemic, including negative impacts on their social health (60%), mental health (44%), the physical health of their close friends and family (33%), their finances (30%), and their employment status (22%). Overall, one-quarter (25%) of Black LGBTQ adults had at least one close friend or family member die from coronavirus illness.

